# Supplementary material for: Are carbon emissions trading and green financial instruments synergistic? -Comprehensive quantitative research based on content analysis
Source: PLoS One. 2024 Mar 7;19(3):e0298601. doi: 10.1371/journal.pone.0298601 (PMC10919601; doi:10.1371/journal.pone.0298601)
Supplement: S1 Data — (ZIP) [file pone.0298601.s001.zip › Data/地级市Z_F工作报告.zip!/║ú─╧/║ú┐┌╩╨/└┤╫╘╣1⁄2╓┌║┼í╛┬φ┐╦╩2╛▌═°í┐_2010.docx]

各位代表：

　　现在，我代表市人民政府向大会报告政府工作，请予审议，并请市政协委员和列席人员提出意见。

　　2009年工作回顾

　　2009年，是我市跨入新世纪以来经济发展最为困难的一年，也是我市积极应对国际金融危机、保持经济社会平稳较快发展、建设最精最美省会城市取得显著成绩的一年。一年来，在省委、省政府和市委的坚强领导下，全市上下深入学习实践科学发展观，认真落实中央和省应对危机一揽子计划，科学谋划，主动作为，全力以赴保增长保民生保稳定，较好地完成了市十四届人大四次会议确定的目标任务。

　　经济保持持续增长。全市生产总值489.6亿元，增长10.8%。全社会固定资产投资277亿元，增长25.1%;社会消费品零售总额277.2亿元，增长18.5%。投资和消费对经济增长的拉动作用更加明显。

　　运行质量稳步提高。财政总收入123.1亿元，增长23.1%，完成年度预算的107%。地方一般预算收入38.4亿元，增长23.9%，完成年度预算的112.5%，税收收入比重达85.6%。驻市金融机构存、贷款分别增长28.4%和42.4%，盈利25.3亿元，增长35.9%。

　　生态环境进一步优化。完成造林2万亩，森林覆盖率达38.3%，比上年提高1.3个百分点。新增文明生态村170个，文明生态村数量占全市自然村总数的62%，比上年提高11.7个百分点。环境目标责任制考核名列全省第一。万元GDP能耗下降3.31%。

　　人民生活不断改善。城镇居民人均可支配收入15237元、农民人均纯收入5643元，分别增长7.7%和8.2%。农村富余劳动力转移就业9112人。城镇新增就业3.59万人，增长14.6%，基本实现零就业家庭动态为零，城镇登记失业率控制在2.5%以内。

　　一年来，我们坚持应急与谋远相结合，主要做了以下六个方面的工作：

　　(一)把保增长放在首位，应对危机成效明显

　　面对复杂多变的经济形势，我们进一步增强经济运行调控的预见性、针对性和实效性，及时采取一系列有效措施，经济增长一季好于一季，回升势头明显。

　　全力以赴扩投资上项目。抢抓中央扩大内需的重大机遇，共争取中央四批新增投资、省配套资金及地方债券9.15亿元，中央新增投资项目总体开工率超过92%;深化与国家开发银行等金融机构的合作，全年政府投资93亿元，增长74.58%。资金投入规模之大前所未有，全年投资对经济增长的贡献率达73.8%。强力推进项目建设，成立“市重点项目办”，落实联席会议制度，建立“绿色通道”。海口港二期、货运大道一期、海新大桥等26个项目顺利完工，海南国际会展中心、马村港一期、电网改造等一批项目加快推进。这些项目既为当前保增长发挥了重要作用，也为海口长远发展夯实了基础。

　　着力扩大消费需求。投入1600万元，开展旅游、房地产和农产品“三大促销”，组织企业到东北、华北、台湾以及东南亚等地举办大型推介活动24场。增加对境外航空公司和组团旅行社的奖励，发放旅游消费券1090万元;房屋销售面积和销售总额分别增长12.9%和32.4%;实现各类农产品订单50.5万吨、总额20亿元，分别增长8.7%和37.9%。落实汽车家电下乡和以旧换新政策，兑现补助资金876万元。有效化解办理房产证历史遗留问题，积极培育旅游、通讯、健身等消费热点，全社会消费需求保持旺盛势头。

　　积极帮助企业渡难关。出台中小企业信用担保、促进房地产健康发展等8项政策措施。建立与各类企业经常性沟通机制。取消房地产中介服务费等8项收费。允许困难企业缓交五项社会保险费，降低失业、工伤、生育保险缴费费率，全市企业少缴三项保险费共3395万元。着力缓解中小企业融资困难，投入担保贷款贴息资金461万元、科技三项费用1430万元;放大融资担保平台的效应，共为288家企业提供11.15亿元的融资支持，分别增长73%和61%。

　　(二)调优扶强特色产业，发展方式加快转变

　　坚持把保增长与调结构有机结合，用足用活国家和省扶持政策，及时出台有针对性的措施，着力推动产业转型升级，特色优势产业稳步发展。

　　积极发展现代农业。安排农林水支出6.8亿元，增长147.9%。加快推进生态循环农业示范区、羊山特色产业带和花卉产业标准化基地建设，畜牧业、渔业、花卉、水果等特色优势产业全面发展。新建标准化养殖小区33个、大中型沼气池13个，新增循环农业示范基地1.32万亩，沼气产业体系建设处于全国领先水平。落实各类惠农政策性补贴4732万元，主要农作物和畜禽良种覆盖率达95%。新成立农民专业合作社226个，全市合作社总数达到470个，带动农户4.5万户，占全市农户总数的34%。电子农务和农技110覆盖全市所有村镇，农产品质量安全合格率达97%。全年实现农业增加值34.1亿元，增长8.1%。

　　扶持发展新型工业。认真落实加快工业发展和促进医药产业、高新技术产业发展等方面的政策措施，成功引进光伏项目，兑现扶助医药产业发展的第45号政府令。海口综合保税区的规划建设和招商选资工作加紧推进，狮子岭工业园、海马工业园、海口药谷、云龙产业园等园区基础设施进一步完善。海宇20万吨镀锡薄板竣工投产，100兆瓦多晶硅太阳能电池、红塔卷烟30万箱易地技改等项目加快推进。我市跨入“国家科技进步示范市”，有国家级企业技术中心4家、省级重点实验室22家、省级工程技术研发中心37家，获得国家各类科技计划项目98个，专利申请量增长24.5%。24家高新技术企业实现工业总产值55亿元，增长24%，高新技术产业发展势头良好。全年工业经济恢复性增长明显，实现工业增加值83.1亿元，增长5.7%。

　　加快发展现代服务业。积极推动旅游业转型升级，推出“印象·海南岛”大型实景演出，大力发展自驾游、高尔夫游、乡村游等旅游新业态。促进旅游与节庆、文体活动的融合，成功举办亚洲沙滩排球锦标赛、中国合唱艺术周等大型活动25个。海口骑楼街区入选首届十大中国历史文化名街。全年接待过夜游客672.3万人次，实现旅游总收入65.01亿元，分别增长5.4%和8.3%。加快发展物流业，开通海口至营口集装箱绿色航线，建成海南花卉大世界、钢材交易市场，开工建设海南中商农产品中心交易市场。积极发展会展经济，全年组织冬交会、旅交会等大型会展62场。住宿餐饮、交通运输、批发零售、金融保险等服务业分别增长7.5%、15%、19.1%、28.5%。全年第三产业完成增加值335.7亿元，增长11.7%。

　　(三)深化改革开放，体制机制创新迈出新步伐

　　认真落实省委、省政府和市委的部署，积极推进改革开放向广度和深度拓展，为我市科学发展增添了新的动力和活力。

　　扎实推进重点领域改革。完成新一轮政府机构改革，突出职能整合，理顺权责关系，共设置工作部门31个，其中新组建部门5个，更名6个，政府机构设置进一步优化。深化行政审批制度改革，实施行政审批大提速，改进机关内部办事流程，强化审批事项监督，审批时限在原有的基础上再压缩一半。规范运作省政府下放的行政管理权项，在全省评比验收中名列第一。农村综合改革和农村土地二次调查基本完成，农村宅基地确权工作全面启动，集体林权制度改革走在全省前列。进一步完善国有资产监管制度，国有资产权益比上年增长53.9%。“海峡股份”成功上市。文化体制改革和医疗卫生体制改革扎实推进。

　　进一步提高对外开放水平。利用外资稳步增长，实际利用外资6.5亿美元，增长15.7%。积极参加第五届珠洽会、中国投资贸易洽谈会等经贸活动，组团到东南亚开展文化交流，区域合作卓有成效。利用海口-东盟对话会平台，发挥海外智囊团的作用，加强对外交流与合作。与南方电网、中国电信海南公司等企业签订战略合作框架协议。

　　(四)完善中心城市功能，城乡面貌进一步改观

　　紧紧围绕最精最美省会城市建设，着力提升城市规划、建设与管理水平，城乡面貌发生很大变化。

　　加强城乡规划和基础设施建设。完成西海岸、海甸岛等17个片区规划，新农村建设规划的编制和试点工作逐步推进。进一步完善城市路网，基本形成“八纵五横”的城市道路框架。长流污水处理厂提前建成，白沙门污水处理厂二期和10座小型垃圾转运站顺利完工。加快基础设施向农村延伸，投入3.93亿元，改造农村公路1124公里，率先在全省实现行政村道路硬化通车;投入4100万元，实施羊山地区农业用水工程;投入840万元，解决32个村庄的饮水安全问题;投入2500多万元，建成23个镇垃圾转运站和400个垃圾收集屋。

　　大力推进旧城改造和新区开发。积极创新投融资模式，提高项目策划水平，改善项目建设环境，吸引更多的社会资金参与旧城改造和新区开发。海甸溪北岸、朱云片区旧城改造进展迅速，共拆迁8419户、93.5万平方米，玉沙村和海甸溪建设安置房6436套，旧城改造稳步推进。第二办公区主体建筑已经封顶，长流新区路网及配套设施加快建设，新埠岛起步区、世纪海港城、鸿洲新城等片区开发顺利推进，新区日渐成形，城市价值明显提升。

　　努力提高城市管理水平。结合新一轮政府机构改革，整合城市管理资源，成立市政市容管理委员会，进一步推进城市统筹管理。大规模开展环境综合整治，投入近3000万元，实行清洁、整洁、规泊、安静、打违“五大工程”，组织25万人次参加环卫大扫除，清理各类沉积垃圾6.9万吨、乱张贴35万多张;取缔占道经营摊点4.11万宗;查处违章乱停车辆5.54万辆次;拆除违法建筑36.8万平方米。整治工作取得明显成效，干净整洁的城市面貌得到社会各界的好评。建立万米网格数字化管理系统，充分发挥街道社区的基层基础作用，初步形成城市管理长效机制。

[nextpage]

　　(五)扎实推进民生工程，社会保持和谐稳定

　　坚持把执政为民的理念转化为保民生的各项具体措施，大幅增加民生投入。全年共投入36.5亿元，增长58.4%，20件“为民办实事”事项基本完成。

　　着力解决民生问题。启动创业型城市创建工作，投入1225万元，帮助困难群体实现就业;把城市环境整治与人性化疏导相结合，设置疏导点101个、摊位6893个，解决了2.1万人的就业。积极推进保障性住房建设，全年廉租房竣工1242套、5.78万平方米，正在建设的有3554套、17.31万平方米;新增经济适用房3710套、38.29万平方米。进一步提高社会保障水平，企业退休人员基本养老金月人均增加110元;调高城镇居民医疗保险支付标准和新型农村合作医疗筹资标准，参合农民住院补偿比例提高15个百分点;安排1961万元，建成4个乡镇敬老院和5个五保之家。切实推进基础教育均衡发展，投入1.48亿元，实施义务教育阶段助学工程，“两免一补”政策得到进一步落实;投入2.14亿元，扩建府城地区中小学校，新增义务教育阶段学位1万多个，学位紧张问题基本缓解;克服困难，设法筹集资金6192万元，积极化解义务教育学校建设的历史债务;启动为期3年、投资9亿元的中小学校舍安全工程;从新增财力中安排1.05亿元，为全市2万多名教师增加岗位绩效工资，实现了教师工资不低于公务员工资水平的目标。完善城乡医疗卫生服务体系，安排5.4亿元，完成市人民医院、中医院、疾病控制中心、“120”急救中心续建工程;投入9600万元，完成乡镇卫生院的扩建改造和设备升级，推进农村和社区卫生服务体系建设，农村卫生室和社区卫生服务网点实现全覆盖;继续稳定低生育水平，人口自然增长率控制在9‰。不断丰富群众文化生活，投入850万元，所有乡镇都建成标准文化站，消除电视广播“盲点村”117个;开展文明生态村千场电影和600场百花奖获奖影片巡演活动。

　　深入推进“平安海口”建设。认真开展以清理侦破积压命案、打击街头“两抢”、打黑除恶、涉毒犯罪为重点的严打整治行动，全市侦破处置积压命案81起，破获现行命案33起，破案率100%，刑事发案率下降9.5%，社会治安明显好转。落实信访工作责任制，成立41个镇(街)矛盾纠纷排查调处中心，妥善处置各类矛盾纠纷5395宗，调处成功率达95%。落实突发公共事件应急预案，有效防范和处理自然灾害和突发社会事件。深入开展“安全生产年”活动，事故起数、伤亡人数分别下降19.1%和8.9%，连续四年被评为全省安全生产先进市。

　　(六)加强政府自身建设，依法行政水平不断提高

　　市政府把“执行力建设”作为深入学习实践科学发展观活动的自选动作，积极推进服务创新、管理创新，加快服务型政府建设。

　　民主法制建设不断加强。自觉接受人大的法律监督和政协的民主监督，定期向市人大报告工作、向市政协通报工作，人大代表的建议和政协委员的提案全部办复。提请市人大常委会审议地方性法规4件，制定和修订政府规章4件。广泛听取各民主党派、工商联、无党派人士意见。进一步抓好政务公开，落实听证制度和新闻发言人制度。推进“市长接待日”常态化，举办“市长与听众面对面”直播活动，加强“12345”政府服务热线建设，全年受理群众诉求2.78万件，办结率98.6%，群众满意率97.7%。深入开展双拥活动，积极支持工会、共青团、妇联、科协、老龄委、关工委开展工作。外事、侨务、对台、民族、宗教等工作取得新成效。

　　“执行力建设”扎实推进。大力开展“执行力建设年”活动，行政审批提速50%，政府执行力和行政效能建设取得明显成效。全面梳理细化政府年度工作，就《政府工作报告》分解的69项任务、与省政府签订的12项责任状事项、20项为民办实事事项以及省委、省政府领导和市委主要领导的批示件，实行严格的督查督办，除调研类的批示件外，其余全部完成。加强行政服务向基层延伸，初步建立市、区、镇(街道)、村(居委会)四级服务体系，群众办事更加便捷。扎实推进“群众满意基层站所”创建活动，在34个部门和937个基层站所开展民主评议。严格行政问责，全年有67名干部因违反工作纪律、执行不力、效率不高被追究效能责任。

　　廉政建设进一步深化。认真落实中央和省关于厉行节约工作的各项要求，全市因公出国出境、车辆购置、公务接待等经费支出的削减数额都超过预定指标。扎实开展“排查防控廉政风险”活动，在52个单位共排查出5466个风险点，建立防控措施8862条。认真治理“小金库”，查缴金额1170.5万元。强化对中央和省扩大内需决策部署的落实检查，加强对政府投资项目的监管，政府部门的廉政建设取得新成效。

　　各位代表!过去一年的成绩来之不易。我们深刻体会到，能够在应对危机中开创新局面，得益于我们坚决贯彻中央应对危机的一揽子计划，不折不扣地把国家和省各项决策部署落到实处;得益于我们把中央的要求与海口的实际紧密结合，科学应对、多策并举，创造性地开展工作;得益于我们把解决眼前困难和立足长远发展相结合，危中求机、化危为机，着力为科学发展蓄能量、打基础;得益于我们坚持以人为本，高度关注民生，确保广大人民群众共享改革发展成果;得益于我们充分调动各方面的积极性和主动性，形成了上下一心、共克时艰的强大合力;得益于市委的坚强领导，市人大、市政协对政府工作的监督和支持。在此，我谨代表市人民政府，向全市人民，向全体人大代表和政协委员，向各民主党派、工商联和社会各界人士，向驻市部队和武警官兵，向所有关心支持海口发展的同志们、朋友们，表示衷心的感谢!

　　在总结成绩的同时，我们也清醒地认识到，我市经济社会发展还存在不少困难和问题：一是经济回升的基础还不稳固，经济总量小、增长乏力仍然是我市最突出的矛盾，保增长的任务还比较艰巨;二是制约发展的体制机制障碍还不少，对外开放的广度深度与特区省会城市的要求还有较大差距;三是旅游资源的开发利用、城市建设管理水平与国际旅游岛战略的要求还有较大距离;四是城乡居民收入增长还不够快，一些事关群众切身利益的问题解决得还不够好，维护稳定的任务比较繁重;五是一些公务员的综合素质还不适应工作要求，个别领域的腐败现象仍然时有发生。这些问题的存在，需要我们采取更加扎实有效的措施，努力加以解决。

　　2010年总体要求和目标任务

　　今年是我市实施“十一五”规划的最后一年，是海南国际旅游岛建设的开局之年。做好今年的工作，对我们继续有效应对国际金融危机影响、保持经济平稳较快发展、为“十二五”规划的启动奠定良好基础，具有十分重要的意义。

　　综合分析国际国内形势，今年经济发展环境好于去年，但面临的形势仍然复杂。一方面，国际金融市场渐趋稳定，我国经济回升向好的基础逐步巩固，中央保持宏观政策的连续性和稳定性，我省大力实施国际旅游岛战略，为我市在新起点上加快发展提供了有利条件。另一方面，国际金融危机的影响还在继续，外部经济环境不确定因素较多，经济增长内生动力不足，新老矛盾和问题交织叠加，保持经济社会平稳较快发展的难度依然很大。我们必须审时度势，积极应对，既要看到有利条件，坚定信心;又要未雨绸缪，把各方面的困难估计得充分一些，努力在新一轮竞争中抢占先机、赢得主动，谱写科学发展、富民强市的新篇章。

　　根据中央和省委、省政府的部署以及市委十一届六次全会精神，今年政府工作的总体要求是：深入贯彻落实科学发展观，把保持经济平稳较快发展、继续推进和谐社会建设作为中心任务，进一步解放思想，更加注重转变发展方式，更加注重体制创新和科技创新，更加注重提升城市品质，更加注重生态文明建设，更加注重改善民生，全力做好国际旅游岛建设的起步工作，努力开创最精最美省会城市建设新局面。

　　今年全市经济社会发展的主要预期目标是：生产总值增长10%，财政总收入和地方一般预算收入均增长15%，全社会固定资产投资增长26%，社会消费品零售总额增长16%，城镇居民人均可支配收入和农民人均纯收入分别增长8%，城镇登记失业率控制在3%以内，人口自然增长率控制在9.3‰以内，万元GDP能耗下降2.85%以上。

[nextpage]

　　围绕今年的目标任务，我们要重点抓好以下八个方面的工作：

　　(一)认真做好国际旅游岛建设的起步工作

　　海南国际旅游岛建设上升为国家战略，中央给予海南从建省办经济特区以来力度最大、范围最广的政策支持。作为省会城市，我市在基础设施、管理、人才等方面处于领先水平，有责任、也有条件发挥自身优势，围绕把海南建设成为“我国旅游业改革创新的试验区、世界一流的海岛休闲度假旅游目的地、全国生态文明建设示范区、国际经济合作和文化交流的重要平台、南海资源开发和服务基地、国家热带现代农业基地”六大战略任务，作出更大贡献。

　　我们一定要深入领会国务院《关于推进海南国际旅游岛建设发展的若干意见》和省委、省政府《贯彻实施〈国务院关于推进海南国际旅游岛建设发展的若干意见〉的决定》的精神，进一步明确海口在国际旅游岛建设中的角色定位，突出优势领域，强化中心城市服务功能，建设全省高新技术产业中心、科教文化中心、会展中心、商务旅游中心、休闲娱乐中心、医疗康体中心和交通枢纽中心，全面打造“宜居、宜业、宜学、宜游”品位之城，努力使海口成为全市人民的幸福家园、国内外游客的休闲乐园。

　　要全力抓好国际旅游岛建设开局之年的八项工作。一是以《海南国际旅游岛建设发展总体规划》为指导，结合“十二五”规划的编制，在城市总体规划和土地利用总体规划的基础上，制定《海南国际旅游岛建设海口市发展规划纲要》;二是研究制定国家赋予国际旅游岛建设相关支持政策的配套措施，争取先行先试，把相关政策落到实处，用好用活;三是培育一批支撑我市推进国际旅游岛建设的重大项目，并争取纳入省“十二五”规划;四是办好游艇经济主题论坛、高尔夫与旅游主题论坛，策划组织一批有影响力的文娱、会展、赛事活动，为我市旅游业的提升创造条件;五是启动对基础设施、交通系统、道路标识、公厕等服务设施的旅游化改造;六是搭建城市公共信息共享平台，策划组织开展一系列高质量的宣传推介活动，积极开发利用火山口世界地质公园、东寨港红树林国家自然保护区等优质旅游资源;七是大力推进保障性住房建设，落实好强岛富民的首要任务;八是打好全民参与的旅游环境整治攻坚战，营造整洁、文明、诚信、和谐的社会环境。

　　(二)强化扩大内需对经济增长的拉动

　　继续抓好国家实施积极财政政策和适度宽松货币政策的机遇，坚持扩大内需，促进投资消费双拉动，加大力度，创新举措，努力保持经济平稳较快发展。

　　促进投资稳定增长。力争全社会固定资产投资完成350亿元，其中市级重点项目69个，年度计划投资207亿元。紧跟国家宏观调控政策的变化，研究国债资金和专项资金支持的重点，抓好环境保护、技术改造、居民住房、教育卫生文化事业等领域的项目策划，力争有一批项目纳入国家和省的投资盘子。进一步深化与金融机构的合作，探索新的投融资模式，积极落实政府投资计划。在扩大投资的同时，注重调整投资结构，鼓励和引导民间资本投向公共服务设施、旅游基础设施和促进消费需求的项目。狠抓重点项目建设，严格落实领导责任制和项目法人负责制，加快海南国际会展中心、国宾馆、长流起步区基础设施等续建项目建设，确保100兆瓦多晶硅太阳能电池、马村港一期等重点项目竣工投产;妥善解决项目用地指标、环评、征地拆迁等问题，积极创造条件，尽快启动国家体育训练基地等一批新项目。

　　努力扩大消费需求。要采取有效措施增加城乡居民收入，健全促进就业创业、劳动者工资增长和支付保障机制，增强城乡居民消费能力。积极引导企业、媒体和社会力量多元联动促销，全方位拓展岛外市场。繁荣城乡消费市场，引导大型商贸企业向新区拓展布局，加强新区现代商业设施和流通网络建设，提高“一镇一集市”、连锁便民店和农资超市覆盖面。继续落实各项促进消费的政策，做好家电汽车下乡和以旧换新、建材下乡等工作。拓展消费新领域，积极培育休闲、康体、演艺等业态，加快发展夜游、夜娱、夜宵、夜购等“夜间经济”。

　　(三)大力发展竞争优势明显的特色产业

　　国际旅游岛建设对产业发展提出了更高要求。我们必须依托资源优势，突出产业特色，调整优化产业结构，努力增强产业竞争力。

　　加快发展以旅游业为龙头的现代服务业。充分发挥海口自然秉赋优越的条件，加强会展、户外有氧运动、康体保健、酒店、历史文化等十大旅游平台建设;制定高尔夫产业和游艇邮轮经济发展规划，积极发展高端旅游;加快推进免税店、综合娱乐城、动漫影视城等一批重大旅游项目的规划建设，启动骑楼建筑历史文化街区保护与综合整治。加快新海物流园、丁村现代美居生活物流园、海南水产物流交易中心和木材交易市场的建设。挖掘琼菜特色资源，引进高档特色餐饮美食项目，引导传统餐饮企业提档升级。培育发展金融保险、中介服务、文化创意等新型业态。

　　加快发展以高新技术为支撑的新型工业。认真落实促进工业发展的各项政策，在项目引进、落户配套、技术研发、政策优惠、综合服务等方面进一步加大扶持力度，推动新型工业加快发展。启动狮子岭二期、海马三期工业园建设，完善各类园区的基础设施，提高企业、项目入园综合服务能力。支持创新型企业、研发中心和重点实验室建设，力争新增高新技术企业12家、创新型企业5家，争创国家创新型试点城市。开展“绿色工业年”活动，重点做好对红塔卷烟30万箱易地技改、椰岛保健酒易地技改和多晶硅太阳能电池二期等项目的跟踪服务。扶持中小企业发展，力争通过担保平台为企业提供12亿元的融资支持。

　　加快发展以生态高效为方向的现代农业。突出“无疫区”品牌，继续抓好生态循环农业示范区、现代农业展示示范园、羊山特色果蔬产业带、三江湾万亩水产养殖示范基地和文心兰产业基地建设。推进海南中商农产品中心和食品工业园建设，打造全省热带农产品集散和深加工中心。积极推广农业新技术，进一步提升农产品的科技含量。加强农产品质量管理，完善农产品质量检测、安全追溯和预警体系，扶持发展绿色、有机农产品。

　　(四)不断增强科学发展的动力和活力

　　加大重点领域和关键环节的改革，大力提升对外开放水平，不断创新和完善科学发展的体制机制。

　　进一步深化各项改革。加大投融资体制改革，创新城市经营方式，优化城投等公司的资产结构，强化投融资功能和资金使用安全。加强国有资产监管，确保国有资产保值增值。深化行政审批制度改革，进一步减少审批环节和审批事项，积极推行网上审批和联合审批，扩大审批大提速成效。加快集体林权制度改革，上半年完成确权发证主体工作任务。全面实施医疗卫生体制改革，重点抓好基本药物制度试点、公立医院改革试点。推进行政事业单位收入分配制度改革，落实事业单位绩效工资(津补贴)制度，完善公务员激励约束机制。加快推进事业单位分类改革，为社会组织发展创造有利条件。

　　大力提升对外开放水平。加快海口综合保税区建设，充分发挥政策优势，努力把保税区建设成为海口对外开放的重要窗口。全方位地开展招商引资，围绕先进制造业、高端服务业、高新技术产业等领域，策划组织系列大型招商引资活动，积极参加省政府组织的各类商贸推介会、项目洽谈会。努力拓展对外交流与合作的渠道和领域，办好第六届泛珠三角省会城市市长论坛;扩大海口—东盟对话会的影响力，进一步发挥海外智囊团的作用。全面优化开放环境，强化部门联动服务机制，完善大通关体系，帮助企业开拓国际市场。

　　(五)切实加快最精最美省会城市建设

　　适应国际旅游岛建设的要求，完善城市功能，优化城市环境，提升城市品质，提高城市文明程度，不断增强省会中心城市的辐射带动能力。

　　提升省会中心城市功能。完善现代交通运输体系。启动马村港二期和新海港建设，配套秀英港基础设施，把海口港建设成为陆岛交通运输集散中心、环北部湾集装箱运输中心港;积极推进美兰机场国际航空楼扩建和第二跑道建设，进一步提升全国大型航空货运枢纽机场地位;完善外部交通与内部路网的衔接，完成东环铁路连接站点建设，加快中线高速海口段、滨海西路二期、龙昆南路延长线的建设。抓住当前岛外需求旺盛、投资加大的有利时机，坚持市区联动、政府主导，积极引进社会资本，加快旧城改造和新区开发，启动滨江新城起步区、镇海村等一批旧改项目，推进长流起步区、新埠岛、江东、大英山等片区开发，进一步改善市民居住条件和城市面貌。坚持公交优先发展，提早规划研究城市轻轨交通布局、构筑城市快速公交通道，进一步优化和新增公交线路，改造新建一批公交场站，恢复建设项目配建停车场功能，着力缓解城市交通和停车难问题。

　　加快小城镇建设。小城镇建设是转移就业、扩大内需、加快新农村建设、提高城市化水平的重要途径。要立足资源条件，依托特色产业，积极推动以10个中心镇为重点的小城镇建设。特别是要围绕羊山地区户外有氧运动和循环经济项目，把永兴镇规划建设成国际旅游小镇和低碳示范镇;围绕云龙产业园和美兰国际机场的配套服务，把云龙镇规划建设成综合服务型小镇。继续开展文明生态村、绿色小康村创建，启动文明生态镇三年建设规划。

　　着力提高城市管理水平。巩固和扩大“五大工程”城市环境综合整治成果，进一步提高城市管理的常态化、规范化、精细化水平。继续完善城市数字化管理信息系统，突出抓好交通秩序、违法建筑等整治。把综合整治与关注民生结合起来，推出利民利市措施，使城市管理执法工作更贴近市民、服务市民、惠及市民。强化属地管理，将更多的资源向基层倾斜，充分发挥街道社区在城市管理中的基础作用。深入开展全国文明城市和国家卫生城市创建活动，进一步提高城市文明程度和整体卫生水平。

　　(六)努力打造绿色低碳城市

　　要加快培育低碳产业，推动低碳技术及产品产业化。编制低碳经济发展规划，逐步把我市建设成为全省乃至全国低碳经济发展的示范区。

　　大力发展清洁能源。抓住中央鼓励发展低碳经济的政策机遇，利用得天独厚的自然条件，积极发展和利用太阳能、天然气、沼气等清洁能源。大力推行太阳能风光互补绿色照明。鼓励引导在各类公共建筑和新建12层以下民用住宅推广使用太阳能热水系统。继续抓好农村沼气建设，进一步完善沼气产业体系，启动低碳农业经济示范区建设。

　　加快推进生态环保工程。坚决淘汰落后产能，大力推广运用节能新技术新产品，建立健全能源消耗检测网络。启动森林城市建设，新增造林1.5万亩，新建滨江西带状公园，完成滨海大道、南海大道美化绿化改造工程。完善长流污水处理厂、桂林洋污水处理厂配套设施和中心城区污水管网，建设医疗废物处置中心，新建一批城乡垃圾转运站，进一步提高污水处理率和垃圾无害化处理率。切实抓好总投资6亿元的水环境综合整治，确保年底中心城区水环境质量明显改善，基本实现水清无异味。

　　实行严格的生态保护制度。坚持把生态环保、低碳经济的理念贯穿于经济社会发展和城市建设的各个领域，完善各项规划，探索绿色发展模式。严格执行环境准入制度，强化节能减排目标责任制，采取有效措施控制主要污染物排放总量，确保全面完成“十一五”减排任务。增强全社会节能环保意识，深入开展节能减排全民行动。

[nextpage]

　　(七)扎实推进以改善民生为重点的社会建设

　　继续将新增财力的55%以上用于改善民生，着力解决人民群众关注的保障性住房、就业、教育、医疗、养老等方面的突出问题，努力让全市人民共享改革发展成果、共享和谐美好生活。

　　扎实推进住房安居工程。下大决心、下大力量，抓住政策机遇，在解决低收入群众、教师、医生、干部职工的住房困难方面，今年要迈出更大的步伐。加大保障性住房的土地供给，抓好廉租房、经济适用房、“双限”商品房的建设，加大城中村、旧城改造及农村危房改造力度，全年计划开工建设保障性住房和政策性住房1.27万套。力争在两三年内有效解决我市中低收入家庭住房困难问题。

　　扎实推进就业创业工程。把推动以创业带动就业、实现再就业工作放在突出位置，力争全年城镇新增就业3.3万人，农村劳动力转移就业1.2万人。深化创业型城市创建工作，扩大小额贷款担保的覆盖面，支持更多的劳动者成为创业者。统筹城乡就业，建立统一规范的人力资源市场。继续加强对下岗、失业人员和农村富余劳动力转移的技能培训，健全城镇零就业家庭、农村低保家庭等就业困难群体的就业援助长效机制，确保全市就业形势基本稳定。

　　扎实推进教育均衡发展工程。深入开展规范化学校创建活动，实施城乡教育对口帮扶，促进城乡义务教育在办学条件、教师队伍、管理水平、生源质量、教学质量等方面均衡发展，力争全市有90所学校达到市级标准，30所达到省级标准。积极实施中小学校舍安全工程。不断提高职业教育水平，加快旅游职业学校、高级技工学校新校区和桂林洋高校区配套建设。

　　扎实推进医疗健康工程。完成旅游医疗服务保障中心建设。统筹利用全市医疗卫生资源，优化基层医疗卫生机构补偿机制，完善社区卫生服务功能，行政村卫生室标准化建设覆盖率达40%以上。完善人口计划生育利益导向机制，继续稳定低生育水平。

　　扎实推进社会保障工程。加快推进新型农村社会养老保险，争取对所有60岁以上农村人口实现全覆盖;探索建立城镇居民养老保险制度。扩大全民医保覆盖面，进一步提高城镇居民医保和新农合补助水平。实行城乡低收入群体生活补贴、城乡低保补贴、城镇低收入住房困难家庭廉租房租赁补贴与价格上涨联动的动态补偿机制，继续提高最低生活保障线。

　　扎实推进“平安海口”创建工程。加强社会治安综合治理，抓好报警与监控系统建设，完善治安防控体系，开展禁毒专项斗争，严厉打击各类违法犯罪活动，为国际旅游岛建设提供安全环境。落实信访维稳工作责任制，加大矛盾纠纷排查调处力度。强化安全生产责任，创新安全生产监管机制，坚决遏止重特大安全事故的发生。加强食品药品安全工作，切实保障人民群众饮食用药安全。完善城市应急管理机制，建成应急指挥中心，提高应对自然灾害、突发公共安全事件的能力。

　　(八)努力建设务实高效廉洁的服务型政府

　　建设国际旅游岛，各级各部门和广大公职人员面临着繁重而艰巨的任务，肩负着光荣而重大的使命。我们一定要以改革创新的精神，强化政府服务，严格落实责任，不断提高行政能力和行政水平，尽心竭力，恪尽职守，努力建设人民满意的政府。

　　全力推进依法行政。把依法行政贯穿到政府决策、执行和监督的全过程，依法行使权力、履行职责。建立健全决策咨询机制，完善重大决策的社会参与、专家咨询、听证和公示制度，推进政府决策的民主化、科学化。认真接受市人大及其常委会的法律监督，坚持定期向市人大常委会报告重要工作。认真接受市政协的民主监督，加强与市政协的工作沟通和协商，主动听取各民主党派、人民团体和社会各界的意见和建议。发挥工会、共青团、妇联等人民团体的作用，加强老龄工作，推进妇女儿童和残疾人事业的发展。继续做好拥军优属、外事侨务、民族宗教、档案史志、防震减灾等工作。

　　不断优化政府服务。继续深入开展“机关效能建设”活动，强化业务培训，提高公务员队伍业务水平和行政执行能力。进一步转变机关作风，坚决克服浮躁心理，沉下心来，打基础、管长远，谋发展、增后劲，要想方设法妥善解决群众、基层的突出困难和问题。创新政府服务手段，建立政府联系企业、服务企业的长效机制。进一步提升政府服务中心、效能投诉、服务热线等平台功能，发挥市、区、镇(街道)、村(居委会)四级服务体系的作用。

　　加强政府廉政建设。认真贯彻十七届中纪委五次全会精神，严格落实党风廉政建设责任制，扎实推进廉政风险防范管理工作。发挥监察、审计的作用，重点加强对建设工程招投标、经营性土地出让、产权交易、政府采购等领域的监管。坚持勤俭办事，科学合理安排财政预算，严格控制一般性支出，确保民生等重点支出需要，加大预算执行力度，提高财政资金使用效益。严格实行行政执法责任追究制，严厉查处执法环节的不作为、乱作为和各种违规违纪行为，坚决纠正损害群众利益的不正之风。

　　各位代表，海口已经迈上新的历史征程，任务更加繁重，前景更加美好!让我们紧密团结在以胡锦涛同志为总书记的党中央周围，在省委、省政府和市委的坚强领导下，依靠全市人民的智慧和力量，进一步解放思想、开拓创新、扎实苦干，为实现“十一五”目标、为加快国际旅游岛建设而努力奋斗!
